# Energy Consumption and Control Response Evaluations of AODV Routing in WSANs for Building-Temperature Control

**DOI:** 10.3390/s130708303

**Published:** 2013-06-27

**Authors:** Apidet Booranawong, Wiklom Teerapabkajorndet, Chusak Limsakul

**Affiliations:** Department of Electrical Engineering, Faculty of Engineering, Prince of Songkla University, 15 Kanjanavanich Road, Kho Hong, Hat-yai, Songkhla 90112, Thailand; E-Mails: wiklom.t@psu.ac.th (W.T.); chusak.l@psu.ac.th (C.L.)

**Keywords:** WSANs, routing, desirable control target, energy consumption, building temperature control system

## Abstract

The main objective of this paper is to investigate the effects of routing protocols on wireless sensor and actuator networks (WSANs), focusing on the control system response and the energy consumption of nodes in a network. We demonstrate that routing algorithms designed without considering the relationship between communication and control cannot be appropriately used in wireless networked control applications. For this purpose, an *ad-hoc* on-demand distance vector (AODV) routing, an IEEE 802.15.4, and a building-temperature control system are employed for this exploration. The findings from our scenarios show that the AODV routing can select a path with a high traffic load for data transmission. It takes a long time before deciding to change a new route although it experiences the unsuccessful transmission of packets. As a result, the desirable control target cannot be achieved in time, and nodes consume more energy due to frequent packet collisions and retransmissions. Consequently, we propose a simple routing solution to alleviate these research problems by modifying the original AODV routing protocol. The delay-threshold is considered to avoid any congested connection during routing procedures. The simulation results demonstrate that our solution can be appropriately applied in WSANs. Both the energy consumption and the control system response are improved.

## 1. Introduction

Wireless sensor and actuator networks (WSANs) comprise groups of sensors and actuators linked by a wireless medium [1,2]. Sensor nodes collect environment information and send their sensory data to actuators via wireless multi-hop communications. Actuators make decisions and then take actions upon the environment based on the available sensor data. Due to the use of actuators, WSANs have ability to change the behavior of environments. Consequently, they can be widely adopted for many applications, including environmental monitoring and control, industrial machine condition, building automation, *etc.* In the building automation application [3–5], WSANs can be utilized to control and maintain the temperature inside buildings. Sensors gather the temperature in the zones of interest and send their signals to the controller. The controller with the control algorithm uses the error between the set-point and the measured temperature values to find an appropriate supply air temperature, and then commands the actuator to perform actions. This procedure continues until the temperature in the zones reaches the desired value.

In WSANs, there are several node constraints in bringing the sensing data from the network to actuators, such as limited energy resources, transmission powers, and radio ranges of the wireless medium. Accordingly, sensor nodes cannot directly send their sensing data to actuators. All nodes in the network must help each other by relaying the sensing data. Routing plays an important role in this multi-hop communication scenario. Due to the limited power of sensor nodes [1,2,6–9], an energy-efficient route setup is required. Nodes should not consume much of their energy resources due to the influence of the routing mechanism [1,10–12]. If sensor nodes cannot transmit the sensing data to the actuators due to insufficient power and the actuators do not update their actions, the desirable control objective will not be achieved. Thus, energy-efficient schemes for prolonging the network lifetime should be considered in WSAN routing design. In addition, the communication among sensors and actuators over unreliable wireless channels can cause the occurrence of packet loss and delay, which degrades the performance of the control system [2,7]. If we neglect the characteristics of the wireless connection, a desired control application will not be working properly. Accordingly, the understanding of the relationship between the wireless network and the control system is required. According to the research literature, as presented in Section 2, most work related to routing protocols for WSANs [13–26] concentrates on either the wireless communication or the control perspectives. Little work has been done on studying the cooperation between the communication and the control [3,4,27]. Furthermore, the energy consumption of sensor nodes as a major concern in WSANs [1,2] has not been yet investigated in the existing works. Based on these knowledge gaps, how to design an efficient routing protocol for WSANs is still an open research issue.

In this paper, we are going to investigate how to design a new routing algorithm for WSANs that can satisfy both communication and control perspectives according to the simulation study. For this purpose, we first study the effects of the AODV routing algorithm on WSANs. We focus on the energy consumption of nodes in a network and the control system response which are the major concerns from wireless networking and control perspectives [2]. Secondly, the proposed routing solution for improving both the energy consumption and the control system response is introduced. The major contributions of our paper include:

- How the AODV routing mechanism impacts the energy consumption and the resulting control performances are investigated. We provide both multi-hop wireless networks and control functions with communication and control performance metrics for studying this issue. We want to demonstrate that the traditional AODV routing protocol designed without considering the relationship between communication and control cannot be appropriately used in wireless networked control applications.
- The proposed routing solution is simple, but it can improve both energy consumption and control system output. Its routing decision is developed based on the information from physical, MAC, and routing layers within the network protocol stack. However, it does not require any modification at physical and MAC layers.

For the first contribution, AODV routing over IEEE 802.15.4 are the communication protocols evaluated in this study. A building temperature control [3-5] is modeled as a control application. Additionally, we intend to generate constant bit rate (CBR) background traffic at different rates on one link of our network scenario. On increasing of the CBR rates, the failure of route establishment process and the possibility of the data packet loss are getting higher. Thus, how the AODV handles this situation and how AODV routing procedure influences control output performances are investigated. The simulation result demonstrates that the original AODV routing algorithm establishes the freshest and the shortest routes for data transmission. It can select a path with a high traffic load although it experiences the high rate of collisions or the unsuccessful transmission of routing and data messages caused by the CBR traffic interruption. The failure of the path-setup and the loss of data messages result in the uncontrollable system and the waste of energy consumption. For the second contribution, we propose a routing solution by enhancing the original AODV routing mechanism to address such research problems. The modified algorithm can automatically avoid any paths with high traffic loads by using a delay-time of a routing message transmission compared to a predefined threshold. On a high-loaded link, messages in queues will wait for a longer time before they are sent in comparison with the case of low loads [28]. Thus, the link is considered to be congested if the delay-time of routing messages is greater than the predefined threshold. The predefined threshold is the average delay of routing message transmissions in the IEEE 802.15.4 wireless network. By this proposed solution, the path with low traffic interruption among possible routes will be often chosen for delivering measured data. This can lead to reduce the failure of path-setup and the loss of data messages. The simulation results also confirm that both the energy consumption and the control system response are improved.

The remainder of this paper is organized as follows: Section 2 discusses related works. Section 3 develops WSAN control system models; the control system model, the WSAN communication model, and the performance metrics are described in details. Section 4 demonstrates the problems of the disjoint communication and control design in WSANs; energy consumption and control response evaluations of the traditional AODV and the proposed routing protocols over IEEE 802.15.4 for building temperature control are investigated. Finally, Section 5 concludes this paper.

## 2. Related Work

As mentioned earlier, how to design an efficient routing algorithm for WSANs that can satisfy both communication and control performances, focusing on the energy consumption of nodes in a network and the control system response is still an open research problem in the current literature. To the best of our knowledge, most research literature related to WSANs focuses on either communication or control separately. There are only a few papers considering the cooperation between the communication and the control in this WSAN research field. Moreover, the energy consumption indicating the lifetime of a network that can significantly influence the resulting control output is not studied in all existing works. A review of related works is given here.

References [13–25] propose routing protocols for WSANs; however, the research methodologies of these studies are still similar to approaches in traditional wireless sensor networks (WSNs). They are only interested in communication viewpoints for designing routing algorithms without considering the functions of controllers, actuators and plants/physical systems. In these works, actuators do not perform any actions to change the behavior of environments. Accordingly, the effect of routing algorithms to the resulting control output is not explored. How these proposed routing protocols can appropriately be applied for control applications is still a research question.

Reference [26] proposes an adaptive protocol for industrial control applications using WSNs. The design approach depends on a constrained optimization formulation problem. The objective function is to minimize the total energy consumption of nodes in a network, while reliability and latency thresholds are the constraints based on control application requirements. The reliability is defined by the probability of successful packet delivery. The latency is described by the probability of largest end-to-end delay less than the threshold. In [26], the reliability must be higher than 90% and the latency must be achieved at least with a probability of 95%. The experimental results from implemented Tmote testbed demonstrate that the proposed protocol outperforms the IEEE 802.15.4 solution. However, the work in [26] only focuses on wireless network mechanisms and doesn't consider the functions of controllers, actuators and plants in the designs and the implementations.

References [29,30] develop a control mechanism for industrial control systems with WSANs where the unreliable wireless transmission plays a major role. A Bernoulli random variable is employed to model the packet loss due to wireless communication among sensors and actuators. Optimal control decisions and the collaboration among actuators are significantly studied in these works. However, these works only focus on the problem of designing a control strategy to achieve control objectives/to meet user requirements. Wireless network protocols that can greatly influence the control system response are not considered in this investigation.

In [3], simulation case studies of wireless networked control systems are presented. The applications considered are the building automation and target tracking cases. The objectives of the investigation are to provide the possibility to study, develop and test integrated wireless communication and control systems by using the simulation tool, namely PiccSIM [31]. This tool is a combination communication and control simulator which is the integration of network simulator version 2 (NS2) and MATLAB/Simulink. Wireless networks are simulated by NS2, while all control parts are simulated by MATLAB/Simulink. Both network protocols and control models are provided to study their performances in this latest work. Additionally, the performance evaluation of localized multiple next-hop routing (LMNR) and AODV algorithms in both control cases are explored. The simulation results demonstrate that the LMNR as the multipath routing can significantly improve robustness in wireless networked control systems. However, any performances in term of the control perspective are not evaluated in the reference [3].

Reference [27] studies routing and control approaches for reducing wireless error-prone effects in WSANs for a mobile robot squad. The objective of this system is to measure and control desired locations of all moving robots. In the control perspective, parameter tuning of a proportional integral derivative (PID) controller and process estimation by a Kalman filter are employed to manage the results of the unreliable wireless communication. To reduce packet loss, multipath routing protocols are evaluated and compared with the AODV in IEEE 802.15.4 wireless networks. An integration of control errors over time is used for the control performance evaluation. We note that the control error is the difference between the actual sensing level and the predefined control target of the physical system. For this case, the actual sensing level and the predefined control target are the actual location and the desired robot location, respectively. The wireless network performance is represented by average end-to-end delay, percentages of packet delivery and routing overhead of the entire simulation time. Reference [27] concludes that the multipath routing is worse than the AODV in mobile WSAN environments. This summary extends the finding from the previous work in [3] where the multipath routing can increase the packet delivery at a sink in stationary WSANs. However, the energy consumption of sensor nodes as a major issue in WSAN [1,2] is not studied in both references [3] and [27].

Reference [4] compares the performance of centralized and distributed control schemes for a building temperature control application over WSANs by the simulation study. The simulation platform is developed using the integration of OMNeT++ tool and MATLAB. A wireless network is simulated by OMNeT++, while control decision making is carried in MATLAB. The network protocols employ the geographical and energy aware routing and the IEEE 802.15.4 protocols. Control errors as the difference between the predefined control target and the measured room temperature are recorded, and the average of the packet loss for the whole simulation is computed for the performance evaluation. However, the major objective of the work in [4] is to develop an appropriate control scheme for WSANs. An exploration of how routing mechanisms affect the desirable control target is beyond the scope of the study.

In [28], an efficient delay-based load-aware on-demand routing (D-LAOR) protocol for mobile *ad-hoc* networks is presented. D-LAOR determines the optimal path by utilizing both the total route delay and the hop-count as the route selection criterion. D-LAOR attempts to avoid any routes with high loads for delivering sensing data by selecting the route with the lowest total delay and the shortest hop-count. Thus, load balancing is achieved by this strategy. However, In the D-LAOR mechanism, each node updates its route entry only when both the total delay and the hop-count from the newly acquired route are smaller than the previous one. By this approach, the shortest route with the smallest total delay may contain a larger load than a route with longer hop-count, as confirmed in Section 5. Having a high load can exhaust a node's resource and increase the possibility of packet loss. Furthermore, the research methodology of the work in [28] is not developed for WSANs. Any control functions are not considered. Accordingly, how D-LAOR can be applied for control applications is not guaranteed.

In [32], the simulation study of the greenhouse climate control system over IEEE 802.15.4 WSANs is introduced. To control the temperature and the humidity inside the greenhouse for the crop growth by using an event-based control approach is the main aim of the study. In the greenhouse control system, sensors gather the temperature and the humidity inside the greenhouse. These collected data are sent to the controller via wireless communications. The controller with the PI control algorithm makes decision and commands the actuators (*i.e*., a ventilator and a heater) to operate their actions. Due to the use of the event-based control approach, each sensor will transmit its sensing data if the value of the difference between the current value of the measured signal and the previous value is greater than a limit threshold. Therefore, an actuator output is only produced when a change is found in the system. The simulation results demonstrate that the event-based control approach can reduce the number of data transmission and can prolong the actuator lifetime. However, the routing mechanism and the energy consumption of a node which can affect the control output are not studied in the reference [32].

Reference [33] develops a decentralized control algorithm for lighting control applications in WSANs. For the proposed decentralized control method, sensor nodes that detect the presence of people in a building first initiate the estimated control signals. The control signal for this case indicates the brightness of lights. After the collaboration among sensors for exchanging the estimated control signals, each node can determine the optimal control signal for controlling the brightness of lights. By this approach, the lights are controlled depending on the response of the occupancy sensors. The occupied areas must be bright and the unoccupied areas should be dark. The experimental results from implemented Mote testbed reveal that the decentralized control algorithm gives the better control performance than the centralized control algorithm. However, the routing algorithm including its effect to resulting control outputs and the energy consumption issue are not investigated in the reference [33].

Reference [34] presents a flexible time-triggered sampling scheme for wireless networked control systems. To adjust the sampling periods of sensors by considering the workload variations is the main goal of the study. The authors claim that the control system may become unstable due to the effects of packet loss and delay in the overloaded conditions. On the other hand, the system performance may be worse than possible due to low resource utilization in the under-loaded conditions. In order to deal with this issue, the actuator in this work will inform the sensor to determine the new sampling period if it does not receive the control commands within a specific deadline time. The simulation results show that the flexible time-triggered sampling scheme can cope with workload variation and can improve the resource efficiency of the system. However, the designing of network protocols for wireless networked control systems are out of the scope of this reference as mentioned by the authors.

In [2], a design methodology for WSANs in mobile control applications is presented. The objective of the study is to develop a simple efficient method on actuators for dealing with unpredictable packet loss caused by unreliable link quality in mobile WSANs. For this purpose, the actuator will perform its actions based on the previous control command values when a sensing data is lost. The actuator calculates an estimate of control command by applying the PID algorithm. We note that the link quality of WSANs in terms of the packet loss rate is extracted from the real experiments; the authors use this packet loss rate information for the simulation study. The simulation results demonstrate that the proposed solution can deal with packet loss; the control performances in terms of the control system output and the integral of absolute error (IAE) are significantly improved. However, how routing algorithms affect the control system response is not studied in the reference [2].

## 3. WSAN System Models

**Figure 1.**
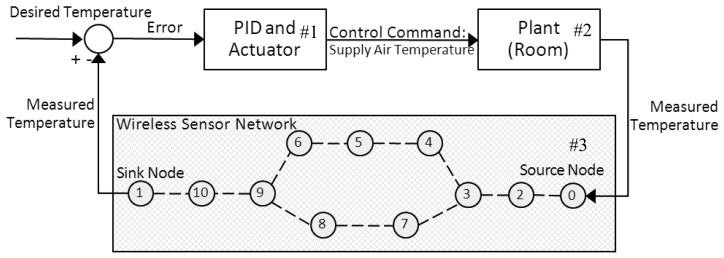
Building-temperature control systems.

The WSAN control application model employed in this work is described in this section. The control application considered is a building-temperature control system as illustrated in Figure 1. To measure and control the temperature of a room over a wireless sensor network is the main purpose of this control system. There are three major components in Figure 1: a controller and an actuator, a plant/a physical system, and a wireless sensor network indicated by block numbers #1, #2, and #3, respectively. The controller is responsible for commanding the heating/cooling actuator based on the room temperature measuring by sensor nodes. The room temperature behavior is characterized by its physical model. In our scenario, the source node ID 0 is placed in the room and detects the temperature every sampling period. The measured room temperature is then encapsulated in a data packet and sent to the sink node ID 1 via wireless multi-hop communications. The controller calculates the error between the desired and the measured temperature values before finding an appropriate supply air temperature command for the actuator. This wireless closed loop control system continues until the temperature in the room reaches the desired target and still has to operate to maintain the control objective. Details of the physical model and the controller studied in our work are described below.

### 3.1. Control System Model

As described before, the temperature behavior in the room is characterized by its physical model. The physical model employed in our study is the zone temperature model. It can be expressed by:

(1) $$H_{a}\frac{dT_{z}}{dt}=F_{sa}\rho_{a}C_{a}(T_{sa}‐T_{z})+U_{\text{Roof}}A_{\text{Roof}}\text{(}T_{\text{Roof}}‐T_{z})+2U_{\text{Wall}1}A_{\text{Wall}1}(T_{\text{Wall}1}‐T_{z})+2U_{\text{Wall}2}A_{\text{Wall}2}(T_{\text{Wall}2}‐T_{z})+q(t)$$

This model is characterized by four state variables which are the supply air temperature (*T_sa_*), the inner roof temperature (*T_Roof_*), the inner wall temperatures (*i.e*., *T_Wa ll_*_1_ and *T_Wa ll_*_2_), and the internal temperature caused by the heat sources [*q*(*t*)]. The supply air temperature is the control input of the model which is determined by the controller and the actuator, while the control output of interest is the zone temperature (*T_z_*). *T_z_* as the latest zone temperature value is collected by the source node at every certain sampling time, and *T_sa_* is immediately updated after the controller finds a new control error. We assume that effects of the East and the West walls on the zone temperature are the same. Effects of the North and the South walls are also considered to be the same. Additionally, we define that two people with load of 150 watts, two lamps with load of 50 watts, and two computers with load of 120 watts are the heat sources causing the warmth of the inside temperature. More details and explanations about Equation (1) can be found in [3,35].

The controller considered in our work is the proportional integral derivative (PID) controller [2]. It is widely used in the wireless networked control process of many applications [2–5]. The PID controller maintains the zone temperature to the desired level by adjusting the supply air temperature based on the control system error. The PID algorithm can be written by:

(2) $$U(t)=K_{p}e(t)+K_{I}\int e(t).dt+K_{D}\frac{de(t)}{dt}(2)$$

where *U*(*t*) is the controller output (*i.e*., the supply air temperature in our case), *K_p_* is the proportional gain, *K_I_* is the integral gain, *K_D_* is the derivative gain, and *e*(*t*) is the control system error between the desired and the measured temperature values. All parameters assigned for the zone temperature model and the PID controller are listed in Table 1.

To evaluate the performance of routing protocols on these WSAN control systems, we conduct a set of experiments using the PiccSIM tool [31]. This tool is an integration of control and communication simulators. Control functions as the zone temperature model and the PID controller are simulated by MATLAB/Simulink, while wireless network communications are simulated by NS2 version 2.33 under Linux operating systems.

#### 3.2. WSAN Simulation Model

**Figure 2.**
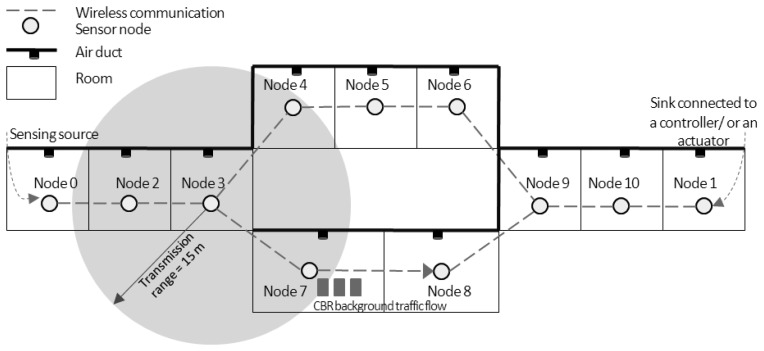
WSAN simulation model.

The WSAN simulation model is shown in Figure 2. Node ID 0 is the only sensing source in our case. This source node detects the temperature in the room and transmits its sensing data to the next node every sampling period. All nodes' locations are fixed and the communication range of each node is not farther than one hop. All radio parameters assigned for sensor nodes are configured according to the IEEE 802.15.4 standard at the 2.4 GHz ISM band. Node ID 1 is the sink node connected to the PID controller/the actuator explained previously. We assume that one sensor node is placed in one room, and the actuator heats the inflow air into the room via the air duct.

In addition, another source-destination pair (node ID 7 and node ID 8) is set for a CBR traffic flow. The aim of this background traffic is to interrupt the data transmissions from the source node ID 0. In WSANs, several nodes may send their routing messages or their sensing data to the sink simultaneously and randomly. This generated traffic can cause more load in the networks and interrupt the data transmissions from any source nodes. The extra CBR traffic load between node ID 7 and node ID 8 is only the illustration of this phenomenon. For this scenario, if temperature data from the source are delivered through the path with traffic interruption, how the AODV routing handles this situation and how the effects of routing procedure influence the control objective are investigated. The CBR traffic has a fixed packet size of 1,000 bytes. We vary the CBR rate from 0.25 Mbps to 3.25 Mbps with a step size of 0.25 Mbps. Node ID 7 initiates to send CBR traffic to node ID 8 when the simulation time is greater than 500 s for all scenarios. To investigate effects of CBR traffic to the communication and the control performances along the simulation time, sensor nodes should not die due to their power constraints. Consequently, we define that all nodes have enough energy to carry on transmissions and receptions during the whole simulation time. All simulation parameters are provided in Table 2.

### 3.3. Performance Metrics

*Communication performance metrics:* The performance measures for evaluating the wireless communication networks are listed as follows: the total number of data packets sent from the source and successfully received at the sink, the jitter duration of data packets, and the total energy consumption of the network.

□ The total number of data packets sent from the source and successfully received at the sink: These metrics are a total number of sensing data packets which carry a value of measured temperature information sent from node ID 0 and successfully delivered to node ID 1.
□ The jitter duration: This metric is the time interval between every two consecutive data packets received at the sink node. It indicates the variation in the delay of successfully received data packets.
□ The total energy consumption of the network: This metric is the sum of all nodes' energy consumptions in all activities during the simulation time. It indicates the energy consumed by a node in the idle listening, transmit and receive states.

Note that for the jitter duration and the energy consumption of the network, we also measure these metrics over a predefined interval of time to study their variations.

*Control performance metrics:* Three metrics are employed to measure the performance of the building-temperature control system: the system output, the settling time, and the integral of absolute error.

□ The system output: This metric represents the zone temperature value in a room at each the given sampling period. This paper plots this value *versus* time to study its time response.
□ The settling time is the time required for the system output to reach and remain within ± 2 % of the desired temperature value. At the settling time, the physical system approaches its steady-state response, which is its approximation to the desired response [37]. If the system output does not reach and remain within ± 2 % of the desired value during the simulation time, the considered physical system is not controllable.
□ The integral of absolute error (IAE) [2]: This measure is the sum of the absolute value of the error between the desired and the measured temperature values defined as
  (3) $$\text{IAE}=\int_{0}^{t}\left|e(t)\right|dt$$
  where *e*(*t*) is the same definition as in the PID control algorithm. The bigger the IAE value the worse the control performance.

Reliability and latency are the important constraints based on control application requirements as discussed in [26]. In this work, the numbers of data packets sent from the source and received at the sink are the measures on the reliability. The jitter duration represents the transmission latency. The energy consumption of the sensor node determines the lifetime of WSANs. The settling time and the IAE are the direct measures of the control system output. This work studies the interdependency among these response variables to gain a better understanding of their importance in an appropriate design of WSNs for control.

## 4. Problem Demonstration of Disjoint Communication and Control Design in WSANs

### 4.1. Traditional AODV over IEEE 802.15.4 for Building-Temperature Control

The AODV routing mechanism is described here. When the source node wants to transmit its data to the sink node, it begins a route establishment process by broadcasting the route request (RREQ) message to all of its neighbors. An intermediate node receiving the first RREQ message sets up a reverse pointer to the source node and rebroadcasts the RREQ. If the intermediate node receives duplicate RREQ messages, it will discard those RREQs. This procedure continues until the first RREQ message reaches the sink node. Upon receiving the RREQ message, the sink node immediately sends the route reply (RREP) message back to the source. An intermediate node receiving the RREP sets up a forward pointer to the sink and forwards the message to the neighbor on the reverse route. After the source receives the RREP message, a data packet is delivered along the RREP path.

#### 4.1.1. Communication Performance Problem

**Figure 3.**
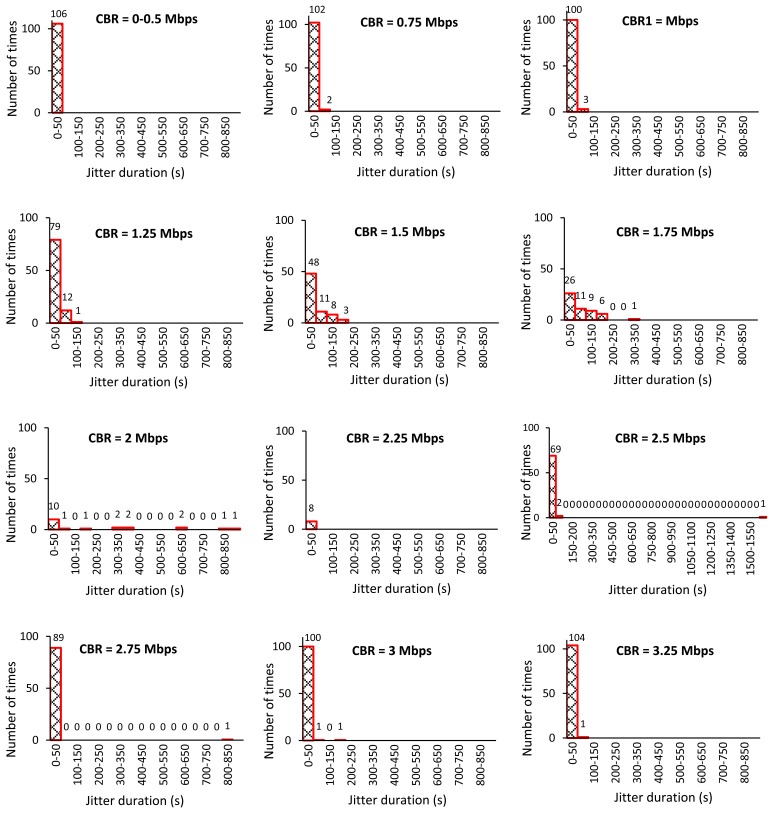
Jitter duration histograms.

The simulated topology illustrated in Figure 2 has only two possible routing paths: upper and lower paths. The upper path consists of node IDs 2, 3, 4, 5, 6, 9, and 10, and the lower path is composed of node IDs 2, 3, 7, 8, 9, and 10. The AODV is a single-path routing protocol, so only the fastest route that RREQ and RREP messages can travel is selected for data transmission. When there is no CBR traffic flow, the lower path is selected. The number of hops in the path indicates the round-trip time in the case of no traffic flow. The AODV determines a route when the source node wants to send its data to the sink as discussed before. The source node establishes a route in every sampling period for our case. Consequently, there is some chance of route changes during the simulation time due to the interruption of CBR traffic flow on the lower path. Table 3 shows the number of data packets successfully sent from node ID 0 and received by node ID 1 *versus* the CBR rate. The jitter duration histograms of all successfully received packets are shown in Figure 3.

From the simulation results, the AODV chooses the lower path for data transmission when there is no background traffic as discussed before. At the CBR rate of 1 Mbps, the AODV often selects the upper path because the CBR traffic flow may interrupts the RREQ message transmission on the lower path occasionally. Due to the hidden node problem, intermediate nodes may not successfully transmit or receive the RREQ or RREP messages. This problem occurs in a wireless network when two nodes that are not visible to each other transmit their packets to a third node that is visible to the formers. This will lead to the loss of the packet [11]. For example, when the CBR message of node 7 and the RREP message of node 9 are delivered to node 8 at the same time, the packet collision occurs due to the hidden node problem. Hence, the source node tries to re-establish the route by sending another RREQ message until the RREP returns to the source. When the CBR rates are increased from 1 to 2.25 Mbps, the number of successfully received packets is getting decreased. Higher CBR rates indicate higher chance of packet collisions. The source node continues retrying to re-establish the route; although, the successful data packets are lower when the CBR is higher.

When the CBR rate is higher than 2.25 Mbps, the upper path becomes faster than the lower path. The AODV changes to select the upper path more often. There is no CBR traffic flow on this upper path; consequently, the communication performance can be recovered on this path. In addition, we have found that on increasing the CBR rates from 2.25 to 3.25 Mbps, CBR packet loss due to buffer overflow at the node ID 7 has occurred at time approximately 2,500 s, 2,000 s, 1,250 s, 500 s, and 500 s respectively. The buffer is full due to the excessive transmission rate, the queue can manage. When the first packet is dropped, all packets arrived after the queues are also dropped. As a result, the amount of CBR packets transmission is largely decreased between node IDs 7 and 8. This will lead to reduce in the packet collisions due to the hidden node problem, especially at node IDs 7, 8, 3 and 9.

#### 4.1.2. Control Performance Problem

**Figure 4.**
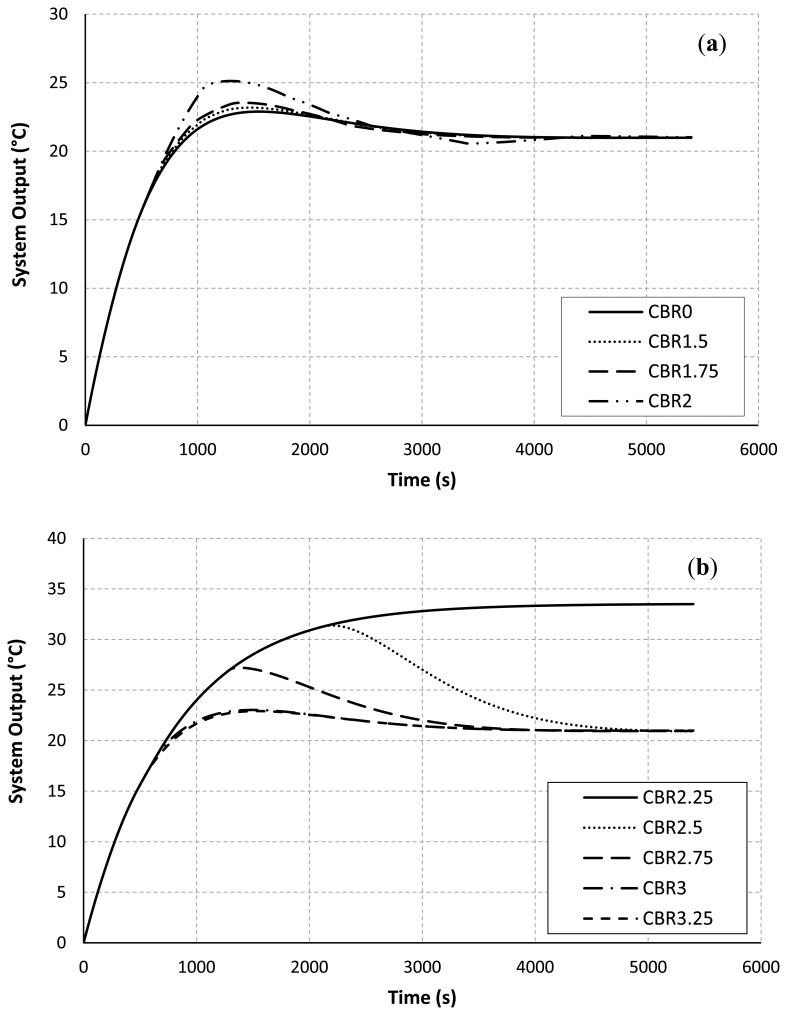
The system output at the plant. (**a**) Vary the CBR rates from 0 to 2.00 Mbps. (**b**) Vary the CBR rates from 2.25 to 3.25 Mbps.

Figure 4 shows the system output from the plant. This plot represents a value of the room temperature sample *versus* the simulation time. The desirable temperature in the room is set at 21 °C. Figure 4(a) shows the system output when the CBR rate is increased from 0 to 2.00 Mbps. The temperature response converges slowly when the CBR rate is getting higher. These results agree on the facts formerly discussed.

In Figure 4(b), the control system is completely uncontrollable at the CBR rate of 2.25 Mbps, and the time response of the room temperature starts to recover at the CBR rates of 3.00 and 3.25 Mbps. This is due to the fact that the route selection is almost completely changed to the upper path as previously discussed in Table 3. Table 4 demonstrates the control performance in term of the settling time.

The results confirm that the CBR rates influence the communication performance and the resulting control output. Four control response cases at the CBR rates between 2.00 and 2.75 cannot converge to the desirable target at the end of simulation time. Why the CBR rates influence the resulting control output is further discussed here. The chances of success in route establishment and temperature-data transmissions are decreased when the CBR rate is high in this scenario. The results of these consecutive communication failures often return worse control performance. When the sink doesn't receive any new data packets for a period of time, the most recent temperature can't be updated at the controller. Consequently, the controller and the actuator cannot feed an appropriate supply air temperature to the room. Thus, the room temperature can't converge to the desired setting point. Consider the results of varying the CBR rate at 2.25 Mbps in Table 3 and Figure 3, there are only nine data packets successfully received at the sink before the beginning of the CBR-traffic flow at the simulation time of 500 s. After that there is no successful data transmission at all. The new control input is not updated for a long period of time. Accordingly, the control response measured by the zone temperature reaches higher than 30 °C as shown in Figure 4(b).

To guarantee an acceptable control performance of the closed loop control system, the sampling period should be set to 4 to 10 times per rise time (*T_r_*) [38–40]. The rise time is the time required for the system response to rise from 0% to 100% of its desired value. Thus, the maximum sampling period or the maximum allowable loop delay [38,39] is equal to *Tr*/4. In our work, the rise time and the maximum allowable loop delay are 900 s and 225 s respectively. Thus, at the CBR rates 0–1.75 Mb/s and 3.00–3.25 Mb/s as in Figure 3, the control system is completely controllable. This is because the jitter durations of successfully received data packets are almost less than 225 s.

**Figure 5.**
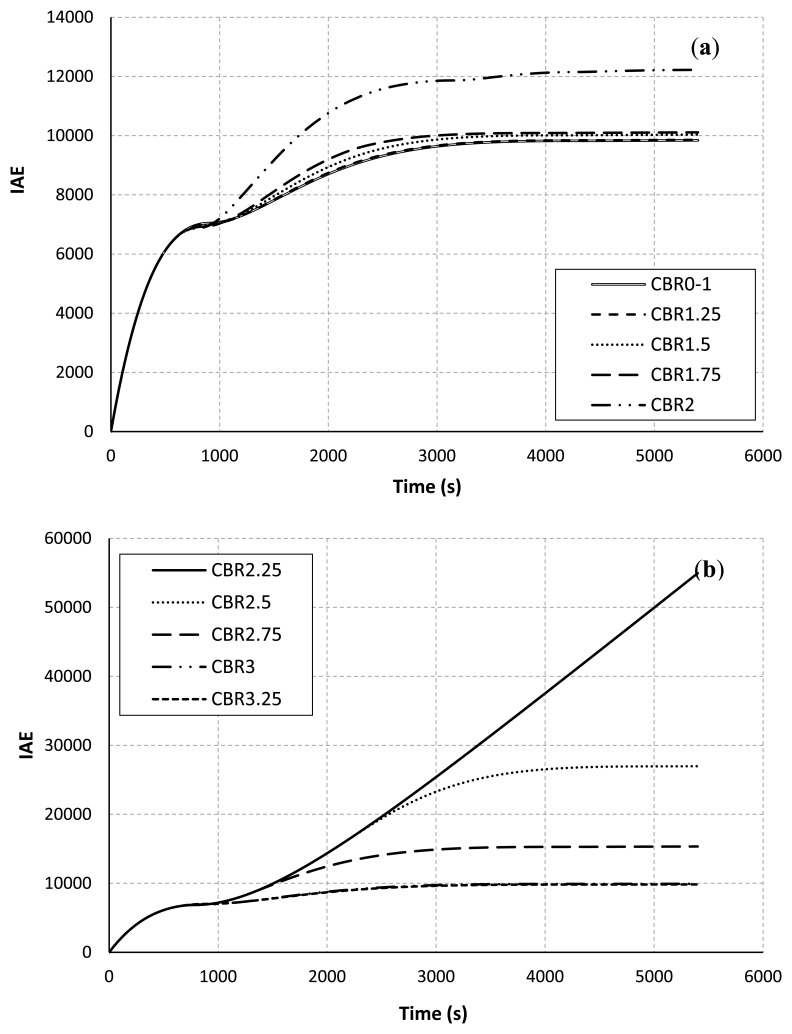
IAE *vs*. CBR rate. (**a**) Vary the CBR rates from 0 to 2.00 Mbps. (**b**) Vary the CBR rates from 2.25 to 3.25 Mbps.

Figure 5 shows the control performance in term of the IAE. The results as in Figures 4 and 5 are directly correlated. This means that when the temperature in the room cannot be controlled to the desired level, the sum of absolute value of the error between the measured temperature and the set-point values is getting bigger. This is because when the measured temperature packets are not reached to the PID controller/the actuator, the controller will not update a suitable supply air temperature and respond to the plant. This will lead to increase in the IAE.

**Figure 6.**
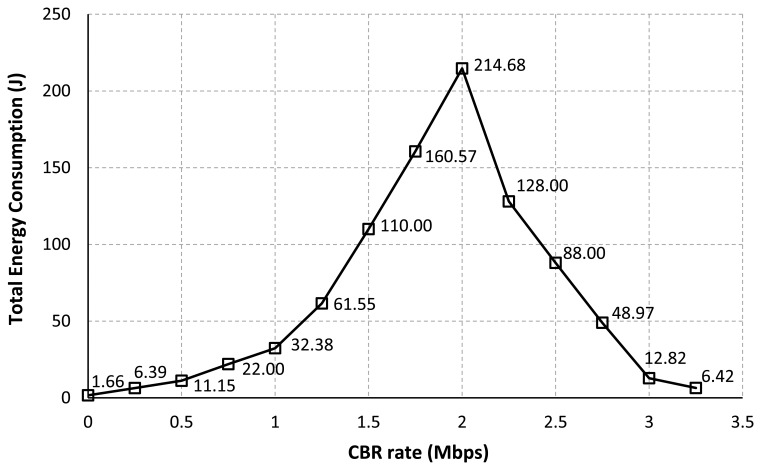
Total energy consumption *vs*. CBR rate.

Figure 6 represents the total energy consumption of all nodes in the network for each CBR rate. The same intuitive explanation as discussed in Table 3 can also be applied for this result. According to the result as in Figure 6, we found that the routing procedure can highly influence to the node consumption. When the process of setting up a route and the data transmission are failed due to the loss of packets, the source will try to re-establish a route by re-broadcasting a new RREO message to the network. More signaling propagated in the network will lead to increase in the energy consumption [11,12]. For our simulation scenario, the number of data packets successfully sent at the source and received by the sink as shown in Table 3 can describe the possibility of a route establishment failure.

#### 4.1.3. Energy Consumption Problem

**Figure 7.**
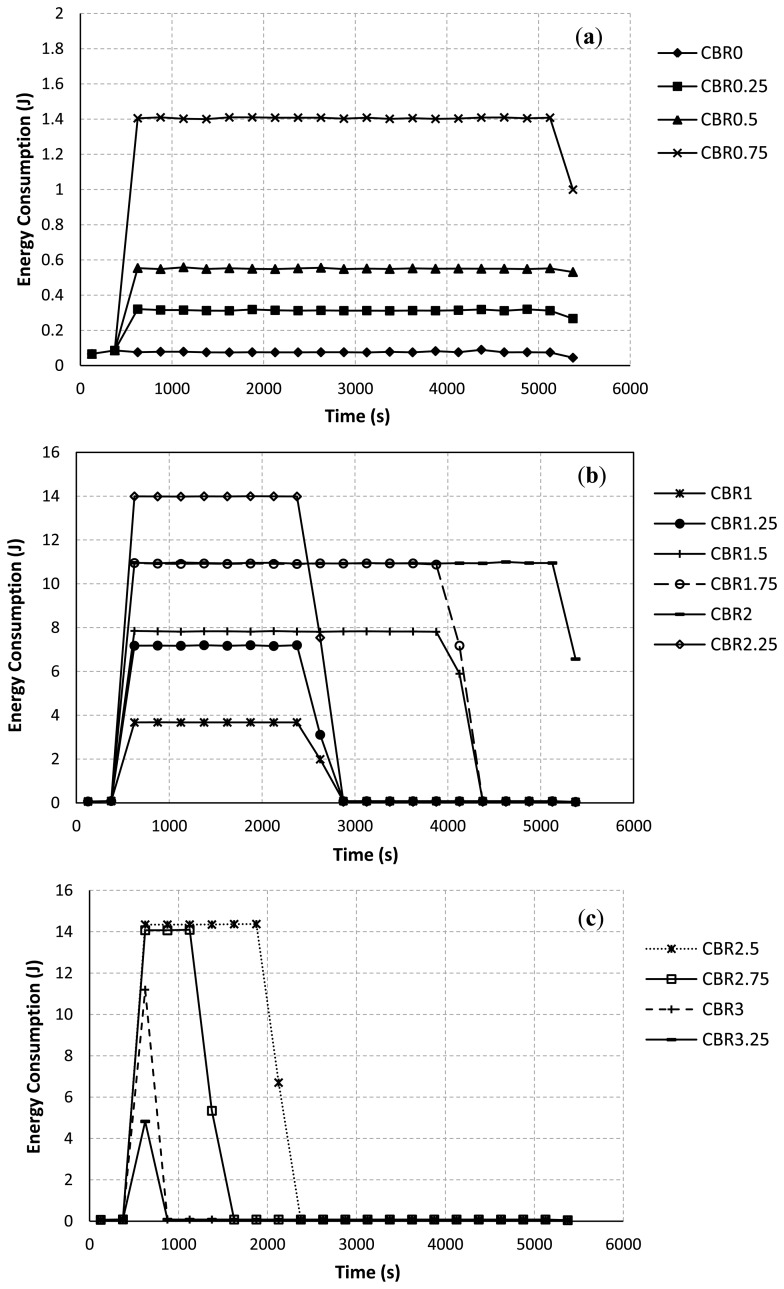
Energy consumption at each window interval *vs*. CBR rate. (**a**) Vary the CBR rates from 0 to 0.75 Mbps. (**b**) Vary the CBR rates from 1.00 to 2.25 Mbps. (**c**) Vary the CBR rates from 2.50 to 3.25 Mbps.

Figure 7 illustrates the energy consumption at each interval of time (every 250 s) during the simulation. The sum of all sample values for each CBR rate in Figure 7 equals to the total energy consumption of all nodes in the network for each CBR rate as in Figure 6. Figure 7 emphasizes that nodes consume much more energy when they try hard to establish/re-establish a route. For example, at the CBR rate of 2.00 Mbps as in Figure 7(b), packet collisions due to the hidden node problem at node IDs 7, 8, 9 and 3 have occurred for a long period of simulation time. The route re-establishment procedure is more repeated at this CBR rate. Therefore, nodes largely consume their power for all intervals of simulation time. At the CBR rate of 3.25 Mbps as in Figure 7(c), the energy consumption is highest at times between 500 s and 750 s. However, route changes after a time of 750 s can lead to reduce the energy consumption of the network. This is because when the upper path is often selected, the probability of the packet collision and the retransmission procedure are getting reduced.

**Figure 8.**
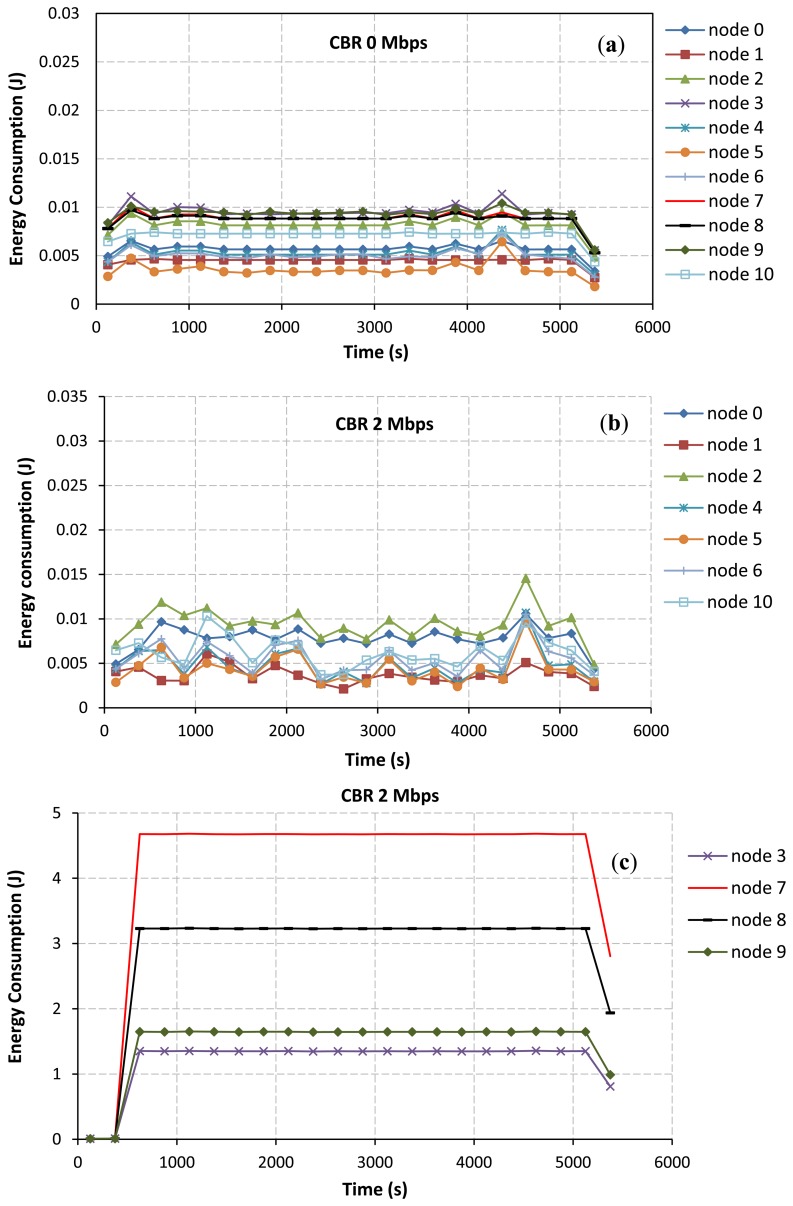

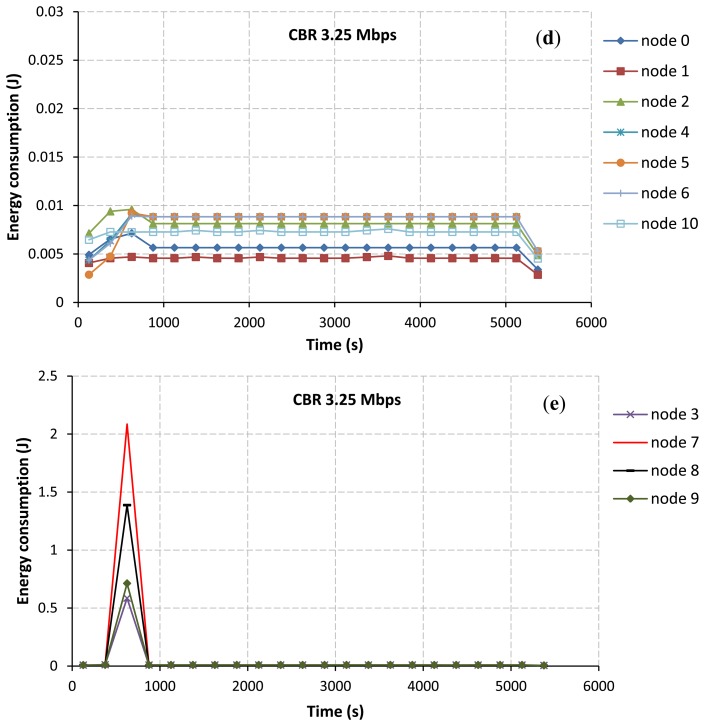
Energy consumption of each node at each window interval *vs*. CBR rate. (**a**) At the CBR rate of 0 Mbps. (**b,c**) At the CBR rate of 2 Mbps. (**d,c**) At the CBR rate of 3.25 Mbps.

In addition, the total energy consumption of nodes directly depends on the varying of the CBR rates at the communication pair between node IDs 7 and 8. When the CBR rate is increased, Node IDs 7 and 8 consume their energies higher than other nodes in the network. Node IDs 9 and 3 as the joint nodes between upper and lower paths also largely use their energies due to the idle listening affected by node IDs 7 and 8. Therefore, the energy consumption of node IDs 7, 8, 9, and 3 will largely impact the total energy consumption of the network. This confirms by some selected results as shown in Figure 8. At the CBR rate of 0 Mbps as in Figure 8(a), node IDs 9 and 3 consume their energies higher than other nodes due to the effects of their locations in the selected path. However, the energy consumption of each node is likely the same at this CBR rate. At the CBR rate of 2 Mbps as in Figure 8(b,c), the AODV routing still tries to select the lower path. Node IDs 7, 8, 9 and 3 consume too much energy, respectively. For this network scenario, if nodes IDs 9 and 3 die due to the lack of powers, the desirable control target cannot be achieved ever. Finally, at the CBR rate of 3.25 Mbps as in Figure 8(d,e), effects of the buffer overflow at node IDs 7 and 8 and the route change mechanism as previously discussed can lead to improve the energy consumption.

From the simulation results in Subsection 4.1, we can summarize that the original AODV routing protocol designed without considering the relationship between control and communication cannot be appropriately applied in WSANs. The control objective cannot be achieved in time, and nodes consume more energy resources.

### 4.2. Modified AODV over IEEE 802.15.4 for Building Temperature Control

In this section, we propose a simple solution by modifying the traditional AODV routing algorithm. The purpose of our solution is to alleviate the research problems discussed in Subsection 4.1, focusing on the improvement of the control system response and the energy consumption. A design concept, a modified operation, and simulation results are introduced as follows.

#### 4.2.1. Design Concepts

As we know from the simulation results described in the previous subsection, the original AODV routing protocol makes the decision to change a route too late. It tries to select the lower path although it experiences the unsuccessful transmission of packets, which results in an uncontrollable system and high power consumption. In addition, the previous results also give the information that route changes can improve both the control output and the energy consumption. The path without the high traffic interruption is the better choice for data transmission. According to this knowledge, a design concept of how the routing algorithm can automatically avoid selecting a path with the high traffic interruption is presented. In this work, a delay-time for the RREQ message transmission between any pair of connected nodes is an indicator used for detecting any congested connection [28]. On the congested link, incoming RREQ messages in buffers will wait for a longer time before they are sent in comparison with the case of no congestion. For this reason, we propose that the considered link is assumed to be congested if the delay-time for the RREQ message transmission is higher than a predefined threshold, and the receiving node in that link will not process the RREQ message further. By this technique, the congested paths will not be selected for data transmission. How the routing algorithm is performed according to the design concept is described below.

#### 4.2.2. Modified Operations

**Figure 9.**
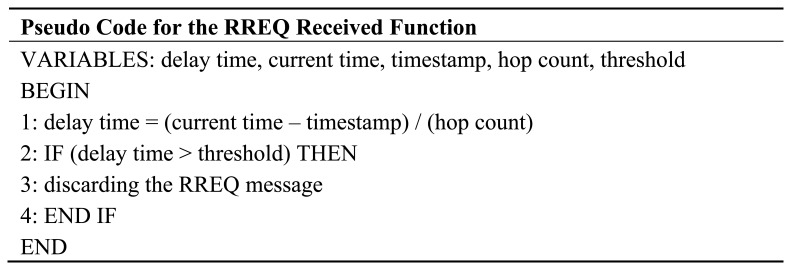
The pseudo code for the RREQ received function. This operation does not require any change in the RREQ message format; therefore, it is able to collaborate with AODV.

We modify the original AODV algorithm in the route request process. Whenever a sensor node receives the RREQ message from its neighbor, the delay-time among them is calculated and compared to the predefined threshold value. The delay-time is the time difference between the current time at the receiving node and the request time (or timestamp) at the source node. It is normalized by the hop-count value which is carried in the RREQ message. Hence, the delay-time in this case is the average value. Additionally, the predefined threshold is the average time that the RREQ message propagates from any sending node at the network layer to any receiving node at the network layer in the case of no background traffic interruption. If the delay-time is greater than the threshold, a node will discard the RREQ message. By using this approach, a relay node can avoid broadcasting the RREQ message to a heavy-load path. Thus, both routing and data messages are frequently transmitted through the path with a low congestion level. Accordingly, this approach can decrease the possibility of the packet collisions occurred in the network as well as the number of route establishment trials. As a result, sensor nodes can save their energy resource larger than using an original AODV approach. How the RREQ message is being processed by a node in the RREQ received function is presented in Figure 9. The threshold can be calculated according to Equations (4–8) and all parameters used for their calculations are defined in Table 5.

(4) $$T_{\text{Threshold}}=T_{\text{Routing}\_\text{layer to MAC}\_\text{layer}}+T_{\text{MAC}\_\text{layer to MAC}\_\text{layer}}+T_{\text{MAC}\_\text{layer to Routing}\_\text{layer}}$$

(5) $$T_{\text{Routing}\_\text{layer to MAC}\_\text{layer}}=0.01\times \{\text{randomvariabla}:\text{uniform}[\text{0},\text{1}]\}$$

(6) $$T_{\text{MAC}\_\text{layer to MAC}\_\text{layer}}=T_{BO}+T_{\text{CCA}}+T_{\text{frame}}+T_{\text{IFS}}$$

(7) $$T_{BO}=BO_{\text{slots}}\times T_{\text{BOslot}}$$

(8) $$T_{\text{frame}}=\left(\text{RRE}Q_{\text{PacketSize}}\times 8\right)/\text{Data}\_\text{Rate}$$

Note that *T_MAC_layer to Routing_ la yer_* in Equation (4) can be ignored since it is very small in comparison with other terms of the equation. *T_MAC_layer to MAC _layer_* in Equation (6) does not include the propagation delay, and all parameters used in this equation are calculated according to the IEEE 802.15.4 standard at the 2.4 GHz ISM band. More details can be found in [41,42]. Note that the threshold set to 0.008840 s as shown in Table 5 may be not suitable for very congested scenarios. In the case that all routes are congested, how to specify an optimal threshold value should be studied further. This work only focuses on performance evaluations and problem demonstrations of routing algorithms in WSANs. The optimal threshold selection for highly congested scenarios is beyond the scope of our current study.

#### 4.2.3. Simulation Results

**Figure 10.**
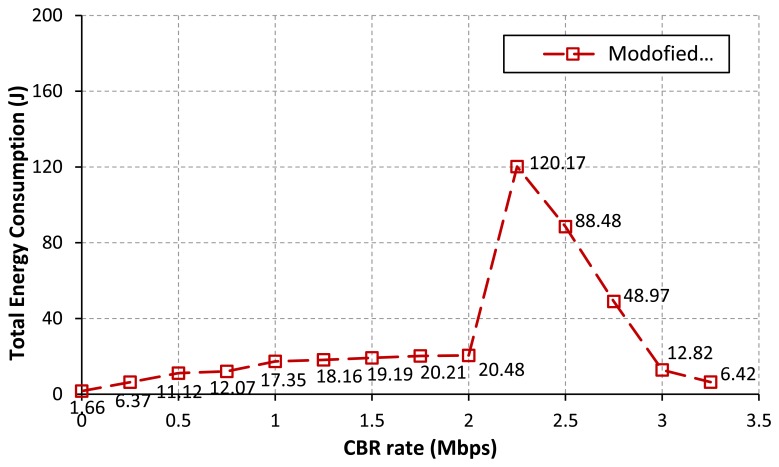
Total energy consumption (modified AODV).

**Figure 11.**
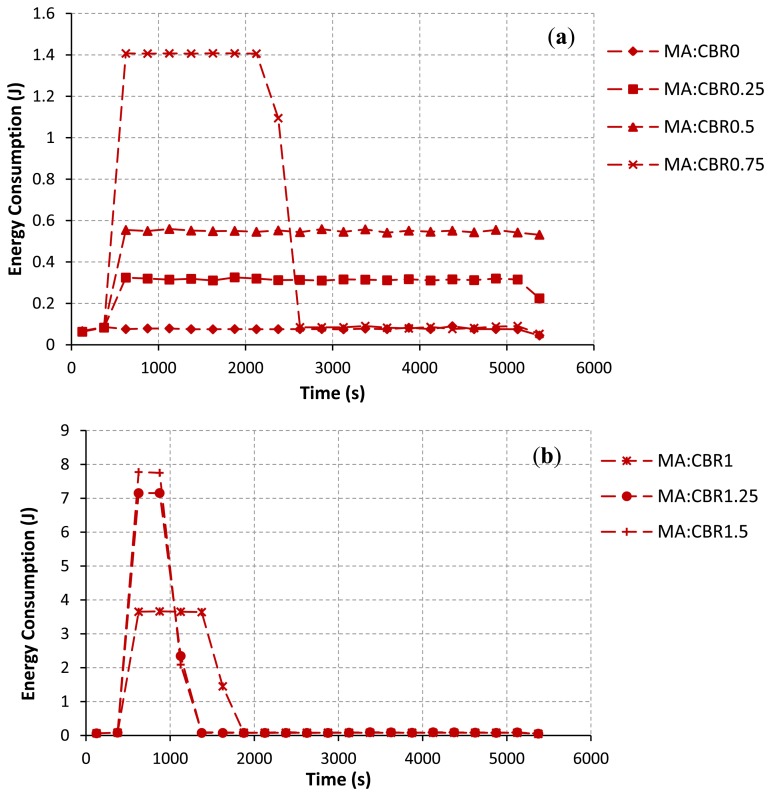

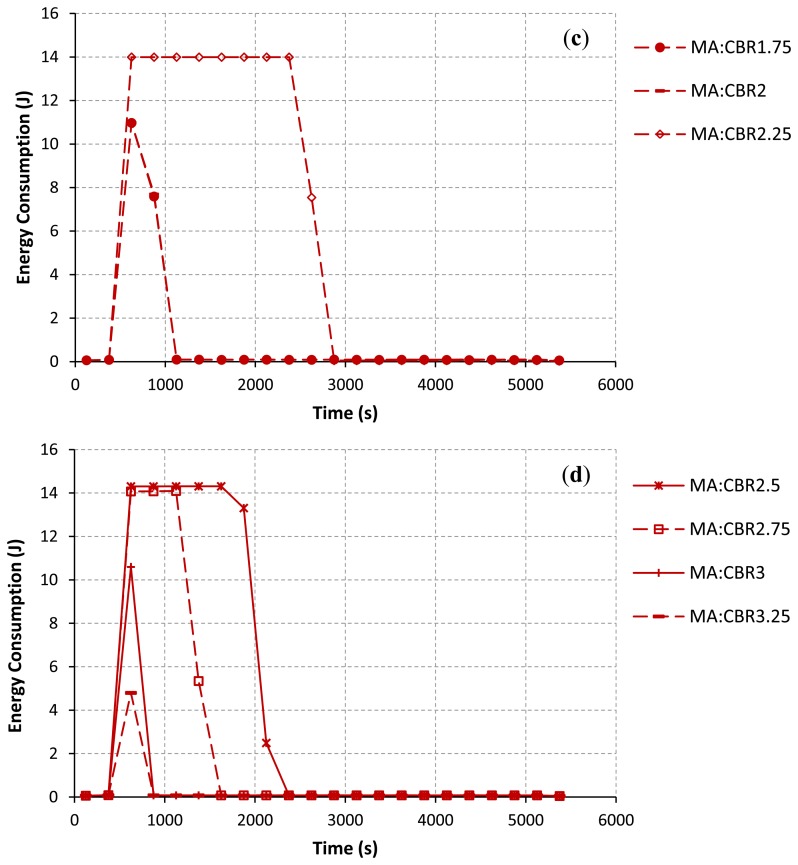
Energy consumption at each window interval *vs*. CBR rate. (**a**) Vary the CBR rates from 0 to 0.75 Mbps. (**b**) Vary the CBR rates from 1.00 to 1.5 Mbps. (**c**) Vary the CBR rates from 1.75 to 2.25 Mbps. (**d**) Vary the CBR rates from 2.50 to 3.25 Mbps.

The simulation results as in Tables 6 to 7 and Figures 10 to 11 show that the modified AODV routing as denoted by MA outperforms the original AODV routing for all response variables. At the CBR rates from 0 to 2 Mbps, the number of data packets successfully received by the sink is getting increased. The settling time is improved, like the case of no CBR background traffic interruption. Additionally, the total energy consumption of nodes is largely decreased. Note that both communication and control performance results are most improved at the CBR rate of 2.0 Mbps. The reason why the proposed solution gives the better results than the AODV is discussed here. According to the proposed algorithm, all nodes in the network avoid to transmit the RREQ message to the path with congestion. Thus, the path with the lower traffic load/ or satisfied the predefined threshold condition is often selected. Delivering routing and data messages along the path with a low traffic load can reduce the possibility of the packet loss and the failure of route establishment process. These can help to improve both the desired control objective and the network performances. For the CBR rates from 2.5 to 3.25 Mbps, the simulation results for all performance metrics are the same as using the original AODV routing protocol. This is because the nature of this network scenario at higher CBR rates and the effect of a buffer overflow influence our proposed solution, like the case of using the AODV routing protocol.

Figure 11 demonstrates that the total energy consumption at each interval of time is reduced when the route changes to the path with a lower traffic load. At the CBR rates from 0.75 to 2.00 Mbps as in Figure 11(a–c), the total energy consumption is getting decreased at time intervals after 2,375 s, 1,625 s, 1,125 s, 1,125 s, 885 s and 885 s, respectively. On increasing of CBR rates, the upper path is fast chosen in comparison with using the original AODV. As formerly discussed, there is no background traffic flow on the upper path; therefore, the level of successful path setup and the successful transmission of data packets are increased. An efficient route setup can directly help nodes to conserve their energies. For the CBR rates from 2.50 to 3.25 Mbps as in Figure 11(d), the simulation results are almost the same as the original AODV routing. The same intuitive explanation as discussed before can be applied for these results.

## 5. Conclusions

This paper evaluates the performance of the AODV routing method on WSANs both from the wireless networking and control point of views. The control application model is the zone temperature model, and the control algorithm is the PID. The AODV routing and the IEEE 802.15.4 standard are the communication protocols. Additionally, we intend to generate CBR background traffic at various rates on one link of our network scenario to study how the AODV handles its route. The process of routing-path setup plays a major role in our performance study. The simulation results demonstrate that the AODV routing takes a long time before deciding to change a route. It remains to select a path with a high traffic load for data transmission although it experiences the high rate of collisions or the unsuccessful transmission of routing and data messages. The unsuccessful path-setup and the loss of data messages increase the total energy consumption of nodes as well as the latency of control convergence time. Our proposed routing solution can alleviate these research problems; both the communication and the control performances are significantly improved.

## Figures and Tables

**Table 1.** Control system parameters.

**Parameters (Unit)**	**Notation**	**Values**

Heat capacity (J/°C)	*H_a_*	89,036.7 [3]
Air density [kg/m^3^]	*Pa*	1.25 [3]
Specific heat capacity of air (J/kg °C)	*C_a_*	1,005 [3]
Room volume (m^3^)	*V_z_*	70.875
Supply air flow (m^3^/s)	*F_sa_*	0.0172[3]
Heat-transfer coefficient of the roof (W/m^2^ °C)	*U_Roof_*	1 [35]
Heat-transfer coefficients of (East, West) walls (W/m^2^ °C)	*U*_*Wall*1_	2 [35]
Heat-transfer coefficients of (North, South) walls (W/m^2^ °C)	*U*_*Wall*2_	2 [35]
Area of the roof (m^2^)	*A_Roof_*	15.75
Area of the wall (East, West) (m^2^)	*A*_*Wall*1_	15.75
Area of the wall (North, South) (m^2^)	*A*_*Wall*2_	20.25
Temperature of the roof (°C)	*T_Roof_*	10
Temperature of the wall (East, West) (°C)	*T*_*Wall*1_	10
Temperature of the wall (North, South) (°C)	*T*_*Wall*2_	10
Power from heat sources (W)	*q*(*t*)	320 [3]
Desired temperature value (°C)	-	21
Sampling period (s)	-	50
Proportional gain	*K_p_*	6
Integral gain	*K_I_*	0.011
Derivative gain	*K_D_*	150

Note: *H_a_* = *C_a_ p_a_V_z_*[3]

**Table 2.** Simulation parameters.

**Parameters Values**
Dimension of the Topology (m^2^) 100 m × 100 m
Simulation time (s) 5,400 s
Number of nodes 11
Radio propagation model Two-ray ground
Interface queue type FIFO with Drop-tail
Interface queue size (packets) 50
Transmission range (m) 15
Initial energy of each node (J) 13000 [36]
Transmit power (W) 0.0744 [36]
Receive power (W) 0.0648 [36]
Idle power (W) 0.00000552 [36]
MAC IEEE 802.15.4
Routing AODV

**Table 3.** Number of data packets successfully sent at the source and received by the sink *versus* the CBR rate.

**CBR Rate (Mbps)**	**Number of Packets Sent at the Source**	**Number of Packets Received by the Sink**
0.00 to 0.50	107	107
0.75	107	105
1.00	107	104
1.25	99	93
1.50	82	71
1.75	69	54
2.00	40	21
2.25	14	9
2.50	78	73
2.75	94	91
3.00	104	103
3.25	106	106

**Table 4.** Settling time *vs*. CBR rate.

**CBR Rate (Mbps)**	**0.00 to 1.25**	**1.50**	**1.75**	**2.00**	**2.25**	**2.50**	**2.75**	**3.00**	**3.25**
Settling time (s)	4,100	4,300	4,650	>5,400	>5,400	>5,400	>5,400	4,100	4,100

**Table 5.** Parameters used for the threshold calculation.

**Parameters**		**Values**

Description	Notation	

Threshold	*T_Threshold_*	8,840 μs
Average time required to propagate the RREQ message from the network layer to the MAC layer at a sender [43]	*T_Routing_layer to Mac_ la yer_*	5,000 μs
Average time required to propagate the RREQ message from the MAC layer at the sender to the MAC layer at the receiver [41,42]	*T_MAC_layer to MAC _ layer_*	3,840 μs
Average time required to propagate the RREQ message from the MAC layer to the network layer at a receiver	*TMAC_layer to Routing_ la yer*	-
Average back off time	*T_BO_*	1,120 μs
Clear channel assessment time	*T_CCA_*	128 μs
Transmission time for the RREQ message	*T_frame_*	1,952 μs
Inter frame spacing (IFS) time	*T_IFS_*	640 μs
Number of back off slots	*BO_slots_*	3.5
Time for a back off slot	*T_BOslot_*	320 μs
RREQ packet size (including Synchronization and PHY headers)	*RREQ_PacketSize_*	61 bytes
Data rate	*Data_Rate*	250 kbps

*Note: T_Routing_layer to Ma c_layer_* and *T_BO_* are set to mean values.

*T_IFS_* is the long-frame spacing time in our case.

Long IFS is used when the packet size is greater than 18 bytes [41,42].

**Table 6.** Number of data packets received by the sink (modified AODV).

**CBR Rate (Mbps)**	**Number of Packets Sent at the Source**	**Number of Packets Received by the Sink**
0.00 to 1.00	107	107
1.25	104	104
1.50	100	100
1.75	97	97
2.00	95	95
2.25	40	37
2.50	78	73
2.75	94	91
3.00	104	103
3.25	106	106

**Table 7.** Settling time *vs*. CBR rate (modified AODV).

**CBR Rate (Mbps)**	**0.00 to 2.00**	**2.25**	**2.50**	**2.75**	**3.00**	**3.25**
Settling Time (s)	4,100	>5,400	>5,400	>5,400	4,100	4,100

## References

[1] Akyildiz I.F., Kasimoglu I.H. (2004). Wireless sensor and actor networks: research challenges. Ad Hoc Netw. J..

[2] Feng X., Yu-Chu T., Yanjun L., Youxian S. (2007). Wireless sensor/actuator network design for mobile control applications. Sensors.

[3] Nethi S., Pohjola M., Eriksson L.M., Jantti R. Simulation Case Studies of Wireless Networked Control System.

[4] Chen J., Cao X., Cheng P., Xiao Y. (2010). Building-environment control with wireless sensor and actuator networks: Centralized *versus* distributed. IEEE Trans. Ind. Electr..

[5] Bjorkbom M., Nethi S., Eriksson L.M., Jantti R. (2011). Wireless control system design and co-simulation. Contr. Eng. Pract..

[6] Gomez C., Paradells J. (2010). Wireless home automation networks: A survey of architecture and technologies. IEEE Commun. Mag..

[7] Feng X. (2008). QoS challenges and opportunities in Wireless sensor/actuator network. Sensors.

[8] Wang X., Vasilakos A.V., Chen M., Liu Y., Kwon T.T. (2012). A survey of green mobile networks: Opportunity and challenges. Mob. Netw. Appl..

[9] Wei G.Y., Ling Y., Guo B., Xiao B., Vasilakos A.V. (2011). Prediction-based data aggregation in wireless sensor networks; Combining grey model and Kaman filter. Comput. Commun..

[10] Xiang L., Luo J., Vasilakos A. Compressed Data Aggregation for Energy Efficient Wireless Sensor Networks.

[11] Chilamkurti N., Zeadally S., Vasilakos A., Sharma V. (2009). Cross-layer support for energy efficient routing in wireless sensor networks. Sensors.

[12] Yen Y.S., Chao H.C., Chang R.S., Vasilakos A. (2011). Flooding-limited and multi-constrained QoS multicast routing based on the genetic algorithm for MANETS. Mathemat. Comput. Model..

[13] Han P., Wu H., Mao D.D., Gao C.S. ELRS: An Energy-Efficient Layered Routing Scheme for Wireless Sensor and Actor Networks.

[14] Boukerche A., Araujo R.B., Villas L. A Wireless Actor and Sensor Networks QoS-Aware Routing Protocol for the Emergency Preparedness Class of Applications.

[15] Chen J., Zhang Y., Cao X., Sun Y. A Communication Paradigm for Wireless Sensor/Actor Networks.

[16] Boukerche A., Araujo R.B., Villas L. Optimal Route Selection for Highly Dynamic Wireless Sensor and Actor Networks Environment.

[17] Boukerche A., Araujo R.B., Villas L. A Novel QoS Based Routing Protocol for Wireless Actor and Sensor Networks.

[18] Garcia M., Coll H., Bri B., Lloret L. Using MANET Protocol in Wireless Sensor and Actor Networks.

[19] Dai Z., Li Z., Wang B., Tang Q. (2009). An energy-aware cluster-based routing protocol for wireless sensor and actor networks. Asian Netw. Sci. Inform. Technol. J..

[20] Guo Y., Xu Z., Chen C., Guan X. (2011). DGR dynamic gradient-based routing protocol for unbalanced and persistent data transmission in wireless sensor and actor networks. J. Zhejiang Univ. Sci. C..

[21] Nethi S., Gao C., Jantti R., Pohjola M. Localized Multiple Next-Hop Routing Protocol.

[22] Barolli L., Yang T., Mino G., Xhafa F., Durresi A. (2010). Routing efficiency in wireless sensor-actor networks considering semi-automated architecture. J. Mob. Multimed..

[23] Dai Z., Wang B., Li Z., Yin A. VDSPT: A Sensor-Actuator Coordination Protocol for Wireless Sensor and Actuator Network Based on Voronoi Diagram and Shortest Path Tree.

[24] Khan M.A., Shah G.A., Ahsan M., Sher M. An Efficient and Reliable Clustering Algorithm for Wireless Sensor Actuator Networks (WSANs).

[25] Chang H.J., Park G.T. Coordinator Assignment Scheme and Routing Algorithm for Wireless Sensor and Actuator Networks.

[26] Park P., Fischione C., Bonivento A. (2010). Breath: An adaptive protocol for industrial control application using wireless sensor networks. IEEE Trans. Mob. Comput..

[27] Pohjola M., Nethi S., Jantti R. (2009). Wireless control of a multihop mobile robot squad. IEEE Wirel. Commun. Spec. Issue Wirel. Commun. Netw. Rob..

[28] Song J.H., Wong V., Leung V.C.M. Load-Aware On-Demand Routing (LAOR) Protocol for Mobile *Ad-Hoc* Networks.

[29] Cao X., Chen J., Gao C., Sun Y. (2009). An optimal control method for applications using wireless sensor/actuator networks. Comput. Electr. Eng..

[30] Chen J., Cao X., Cheng P., Xiao Y. (2010). Distributed collaborative control for industrial automation with wireless sensor and actuator networks. IEEE Trans. Ind. Electr..

[31] Kohtamaki T., Pohjola M., Brand J., Eriksson L.M. PiccSIM Toolchain-Design, Simulation and Automatic Implementation of Wireless Networked Control Systems.

[32] Pawlowski A., Guzman J.L., Rodriguez F., Berenguel M. (2009). Simulation of greenhouse climate monitoring and control with wireless sensor network and event-based control. Sensors.

[33] Masayuki N., Atsushi S., Jiro N. (2009). Distributed environment control using wireless sensor/actuator networks for lighting applications. Sensors.

[34] Feng X., Wenhong Z. (2007). Flexible time-triggered sampling in smart sensor-based wireless control systems. Sensors.

[35] Tashtoush B., Molhim M., Al-Rousan M. (2005). Dynamic model of an HAVC system for control analysis. Energy..

[36] Jin C., Kunz T. (2011). Smart home networking: Lesson from combining wireless and power-line networking. Smart Grid Renew. Energy J..

[37] Nise N.S. (2004). Control Systems Engineering.

[38] Ji K., Kim W.J. (2008). Optimal bandwidth allocation and QoS-adaptive control co-design for networked control systems. Int. J. Contr. Autom. Syst..

[39] Bai T., Wu Z.M., Yang G.K. (2005). Optimal bandwidth scheduling of networked control systems (NCSs) in accordance with jitter. J. Zhejiang Univ. Sci..

[40] Bjorkbom M. (2010). Wireless Control System Simulation and Network Adaptive Control. Ph.D. Thesis.

[41] Latre B., Mil P.D., Moerman L., Dierdonck N.V., Dhoedt B., Demeester P. (2005). Maximum throughput and minimum delay in IEEE 802.15.4. Lect. Note. Comput. Sci..

[42] Latre B., Mil P.D., Moerman I., Dhoedt B., Demeester P. (2006). Throughput and delay analysis of unslotted IEEE 802.15.4. J. Netw..

[43] Perkins C., Belding-Royer E., Das S. *Ad-hoc* on-Demand Distance Vector (AODV) Routing (RFC 3561). http://www.ietf.org/rfc/rfc3561.txt.

